# Dr. Kinglake, in Reply to Messrs. Wayte and Atkinson on Obstetric Practice

**Published:** 1816-03

**Authors:** 


*For the London Medical and Physical Journal.*

### Dr. Kinglake, *in Reply to* Messrs. Wayte *and* Atkinson, *on Obstetric Practice.*

YOUR correspondent Mr. Wayte is entitled to my respectful acknowledgment of his liberal and intelligent comments on my opinion relative to ordinary man-midwifery practice. It would have been difficult for any person to have remarked more appropriately and pointedly than Mr. Wayte has done: it, therefore, becomes my duty to state why observations so exemplarily critical on the subject fail in compelling me to acquiesce in them. I have taken my main stand on the grand basis of natural provision,—a stand that must be regarded, in all questions of natural philosophy, as the *vantage ground* of truth.

It is not to be conceived that an occasion so directly connected with, and so immediately involving, the continuance of human life, could be left so exposed to danger as to have rendered it doubtful whether the unassisted pains and perils of birth might not, in most instances, prove destructive both to the female and to her offspring. Had the human animal œconomy been constructed with such deadly liabilities at the period of being ushered into the world, random indeed, of the most fortuitous kind, might justly be said to have prevailed in the organization of animal nature, rather than the exquisite design, contrivance, and adequacy, that beautifully pervade every portion of it. To gravely affirm that Nature's provision for parturition is too insufficient to be safely left to its own resources, appears to me to be as far-fetched, and as untenable, as it would be to affirm that the peristaltic power power of the intestines is not equal to the due expulsion of their excrementitious contents; that the urinary bladder cannot sufficiently contract to discharge its enclosed fluid; nay, that the heart itself, with its extensive and various vascular appendages, could not adequately act without the aid of art. The accidental insufficiencies of Nature are not her original handy-work: they are, in every instance, morbid deviations from the primitive perfection under which she invariably appears.

Admitting, then, what cannot be denied, that Nature's works are equal to all their ends, and that it is with the deviations from the natural standard of perfection that the hand of art has to do, I will cursorily examine the various instances in which your respectable correspondent has contended for the employ of scientific man-midwifery; and it will then be seen whether he has substantiated his occasions for the practice in a way that would not only render its adoption popular, but even philosophically vindicable.

With regard to the placental presentation, said to occasionally occur, it appears to me that the occasion is as rare as a deviation from the natural course can be; indeed, instead of a deviation from, it may be said to be an inversion of, the order of nature, which is a topsy-turvy course not reasonably to be calculated on. A mal-formation of the heart, of the intestines, or of any other viscus, is as likely to happen, as for a placenta to be attached over the orifice, instead of at the bottom, of the uterus. If my information be correct, it is not risking too much to say, that there is not more than one praсtitioner in a thousand in any age, in any country, that has ever met with an instance of it;—and is the bare possibility of such an occurrence, amidst the extreme rareness with which it has actually taken place, to be one of the leading grounds on which indiscriminate man-midwifery practice is to be founded? But, admitting it happened often enough to excite an apprehension in every case that it might occur, may not female midwives be sufficiently instructed at least to know the exigencies of the case, if not capable of affording the requisite aid, so that the competent practitioner may be as seasonably and as availingly called to assist in that emergency, as he could in other cases of dangerously bleeding vessels, fractures, &c. Nor is it clear that a placental presentation unrelieved, and unperforated for the manual delivery of the fœtus, would terminate in death. The uterine contraction that would detach a placenta from the os uteri, would also advance the head of the fœtus sufficiently close to the bleeding source, to restrain, by the firm pressure it would occasion, the effusion of blood

 within within safe limits. This presentation, therefore, in addition to its being much too unfrequent for anxious calculation, might actually occur, and terminate favourably without the intervention of art.

Your correspondent asks, "Must we not, at times, assist in dilating a rigid os uteri?" To which my reply is, that, with all due deference to his experience in obstetric practice, I know not the occasions in which such interference with the parturient action of the uterus is at all necessary; and it happens to be one of the instances that was in my view when I employed the terms *intermeddling*, *mischievous*, &c. I think that any sort of interference with the excitability of the impregnated uterus, in its closed state, may be likely to derange and injure the natural process of parturition; and the circumstance of rigidity in the os uteri denotes that the secreting action of the part is not sufficiently advanced for the expulsion of the fœtus. I have, therefore, in such instances, regarded and denominated such affectation of aid as gratuitous, meretricious, busy, hurtful, &c. These epithets may be deemed harsh, but the cause in which they are used is that of philosophical truth, which disdains blandishments, and expresses itself in unequivocal firmness.

The requisite assistance claimed by your correspondent in a face-presentation, for the purpose of shortening its axis, is, in my judgment, refining too much on the variable posture of the presenting part of the fœtus, during uterine contraction, to be of practical utility. Slight deviations from the right line of exclusion are rather the momentary effects of the contractile action exerted on the presenting part, than a stationary position of the protruding portion of the fœtus. The attempt here also to interfere, I would very respectfully say to your correspondent, would be rather an hurtful intermeddling than a beneficial practice.

Profuse uterine hæmorrhages after delivery are also among the objects on which your correspondent rests his defence of man-midwifery. These are afflicting occurrences, but, perhaps, more so in aspect than reality, because the concomitant danger is much less than is often imagined from the salutary circumstance of the hæmorrhage curing itself by the alarming extent of the effusion. When the contents of the sanguiferous vessels are greatly and rapidly diminished, a state of comparative inaction of the heart and arteries is induced, approaching to syncope: during this state, the blood is not propelled with sufficient force to carry on the effusion, when the leaking sources of it become plugged by *coagula*, that happily restrain any further hurtful escape of the sanguineous fluid. What better, or rather what so well, well, could be done by an obstetric practitioner? No stimulant medicine would be admissible, no manual aid could be usefully offered. Low temperature, and the recumbent posture, constitute nearly the whole assistance that this popularly terrifying scene of blood, and, as it were, suspended animation, would seem to require.

Why the practice of midwifery should have passed from the hands of women into those of men, your correspondent must know is rather a question of history, and of the moral circumstances of society, than one of " reason" or of " necessity." The *necessity* that your correspondent would insist on is but of modern date: female parturition must have existed over the habitable surface of the globe ever since the commencement of human nature. Why then should this imperious necessity have been so slow in proclaiming itself? The female system has always been what it now is; and the population of remote antiquity was much more numerous than it appears to be at the present time. The issue into life at those periods of antiquity appeared to be unencumbered by the obstacles that your correspondent recites; at least, we know that there was no one so skilled in the obstetric art as either to detect or remove them. What sort of *necessity*, then, is this which your correspondent assumes for midwifery to pass from the hands of women to those of men? It pleads neither antiquity, nor any ancient disadvantages from its not having been known and earlier adopted: it would seem, therefore, to be a *necessity* rather of artificial than of natural creation, and is, of course, open to the imperfection of all human devices. The " strong reason" that your correspondent speaks of as the foundation of his assumed *necessity*, rests on grounds quite as unstable as those of the *necessity* itself: in short, both the one and the other appear to be derived rather from professional *craft*, as formerly intimated, than from a philosophical persuasion of its *necessity*.

Your correspondent has, in a way unworthy of his intelligence, enlisted into the number of his arguments for the practice of man-midwifery, an assertion that there is not one woman in a hundred who would not prefer a male practitioner on midwifery occasions, because they think themselves more safe! This instance of popular preference on the part of females towards male practitioners in midwifery proves the incorrectness of appealing to them on the subject, and shows, indeed, the trade alacrity with which female prejudice has been cajoled and confirmed on the occasion. This proceeding may be consistently stated as illustrative of the rise and progress of man-midwifery practice, but it cannot be admitted as any sort of explanation why, in justice and in truth, it ought to have been transferred from the female to the male hand.

Your correspondent, after having exhausted the graver part of his reasoning on the *necessity* of man-midwifery, condescends to triteness, bordering on ridicule, in putting the question—"Does the doctor think women preferable, through their ignorance, and inadequacy to difficulties; or men improper, because well versed in the whole department? Ignorance is surely much more prone to blunder, than science to be over officious." It was not to be expected that a person of your correspondent's apparent acquirements would have dealt in mere expletives: nothing can be more pointless and inapplicable than the doctrine of contraries in a scientific discussion. Occasions do present where the severity and felicity of argument may be relieved by judicious sarcasms; but your correspondent is not entitled to that latitude of indulgence. He must previously explain the claims of man-midwifery for general adoption, on principles and facts much more convincing and conclusive than any thing he has adduced in his paper under consideration.

My object in publishing my late remarks on the dispensable claims of the obstetric art was to place under the competent direction of Nature an office as absolutely and unexceptionably provided for as any other function in the animal œconomy. I do therefore contend that the practice of medical accoucheurs attending in all cases of parturition, lest something might arise requiring scientific aid, is not less preposterous than incessantly attending on a person lest the liver should not secrete bile, the kidneys urine, or any other viscus should cease to perform its peculiar function.

The diseases of females connected with utero-gestation very naturally fall within the province of medical treatment, as do all the deviations and irregularities during the parturine effort under the skilful and scientific accoucheur; but were the ordinary practice of midwifery to be confined to women, and reference only had to a male practitioner in cases of absolute necessity, much less would be heard of preternatural labours, laborious and inefficient efforts for natural parturition, and of resorting to manual and instrumental aid. Undisturbed nature would then proceed slowly, safely, and efficiently, for the seasonable expulsion of the fœtus. No violence would have been in any way added to the usual excitement of the uterus; and though, in some cases, the period of parturition may seem to be prolonged, yet it is more than probable that the delay will be abundantly compensated by the unimpaired health of both mother and child, child, by freedom from inordinate after-pains, from puerperal disease, from uterine distemper, and various other affections, growing out of, or associating with, a morbid derangement of the female genital system; from infantile mutilation, deformity, and various other irreparable injuries.

It is not my object, nor would my feelings permit me, to do ample justice to my firm persuasion that mischiefs of the most calamitous nature result from the present indiscriminate and extended scale of man-midwifery. To say nothing of the unquestionable instances of mal-formation of the pelvis, of a monstrous and impassable volume of head to descend through that bony boundary, it cannot be heard, without shuddering, that the practice is not rare in which, after a lapse of less than twelve hours in lingering and inefficient labour-pains, the prompt, the skilful, the instrumental accoucheur denounces the sufficiency of Nature; and, where the presentation is natural, where no symptoms of imminent danger on the part of the mother have arisen, he commences his scientific work by boring the fœtal skull, and compressing it within practicable limits for extraction; and when the ill-judged destructive interference is over, full credit is asked, and given, for having saved the mother's life; and the *obstetric warranty* is then gravely pronounced—*when difficulties compel either the sacrifice of mother or child, the latter is the authorised victim!* Such cases have occurred, are occurring, and will continue to occur, as long as man-midwifery shall continue to be practised as universally as it is at present, and with all the gravity and grimace of its being indispensably necessary.

The cases which are now, to an incalculably baneful extent, falling under the superior adroitness of the skilful obstetrician, and which must be handled and managed with the instrumental tact of his erudite art, would, I verily believe, for the most part, prove common and favourable occurrences in the uninterrupted course of nature. The cause of humanity is deeply concerned in the utter abandonment of ordinary man-midwifery; in leaving natural labour to its own resources, soothed and cherished only by the benevolent kindnesses of female attendants. By this happy reclamation of natural right, the inherent sufficiency of the parturine function will be ascertained and established. The artificial incapabilities of nature will no longer be recognised; and the real occasions for scientific accoucheurship will be too seldom either to awaken female dread, or to countenance the watchful calculations of the obstetric theorist.

After the above was written, your Journal for the present

 month month reached me, in which I observe some remarks, published by Mr. ATKINSON, on my opinion relative to obstetric practice. Mr. Atkinson has gone more into practical detail on the subject than Mr. Wayte has done; and it may be justly added, that he has very candidly and intelligently stated his arguments in behalf of the prevailing system of man-midwifery: I wish I could say that he has been more successful in convincing me of the truth and conclusiveness of his positions. The various reasons he has adduced for authorising the unaltered continuance of the practice, lose, in my judgment, all their weight, when it is considered, that those reasons, as well as others that might be offered, rest on *exceptions* to a general rule, rather than on the legitimate demands of nature. To argue from exceptions may be plausible; but the stability of the general principle cannot be invalidated by it.

My objection to uniform man-midwifery practice is founded on the corollary that Nature is fully equal to her own work, and cannot be advantageously assisted in the execution of it. To speak of exceptions or deviations from this inherent correctness, is rather to imagine than satisfactorily to exhibit proofs of such insufficiency. It would be easy to conceive an endless extent and variety in the obstacles to the natural efficiency of power, yet it would be extremely difficult to state them in a way that could bring them under the regulation and controul of art. In the course of nature, with reference either to the animal economy, or to the general laws of motion, do impediments present often enough to need the countervailing and amending aid of human art? Animals live, the planets revolve in their respective spheres, repulsion and gravitation, in their infinite modifications, proceed with too much of the undeviating precision of primordial perfection to require the interposition of human assistance to rectify and adjust incidental irregularities.

The list of claims cited by Mr. Atkinson for indiscriminate man-midwifery practice would, if well-founded, fully justify what he so strenuously insists on; and the bare enumeration of such a groupe is sufficient to terrify the unprofessional and the prejudiced into a persuasion that the practice could not, in any case, be dispensed with. But what is the real fact? Why, it has been ascertained to an extent that sets all questions at rest on the subject, that medical practitioners, in full midwifery employ during upwards of thirty years, have never met with an unnatural presentation, have never had an occasion for using an instrument, and have always found the natural efforts equal to all the exigencies of salutary parturition. This demonstrative proof of the undue zeal zeal with which the alleged indispensable necessity of uniform man-midwifery is urged, might be further verified by the names of practitioners that would render it incontestible; but is not the same affirmed, trumpet-tongued, and beyond contradiction, by the notorious fact that the Asiatic, the African, and the uncivilized part of the American females, to this hour, are for the most part left to spontaneous parturition, and the historians of those people have not cited any of them as instances of suffering for want of the obstetric practice!

Nature possesses much pliancy, and may be variously modified for purposes of art; but this facility of adaptation should not be mistaken for an inherent defect requiring to be supplied by artificial means. Any disease incident to the human body is constantly more likely to occur than a deviation from the natural course of parturition requiring manual aid; and would it not be deplorably absurd to be in attendance on a person lest a disease at any moment should arise?

The baneful effects of man-midwifery practice in every description of case are the popular expectation that something is to be done where nothing ought to be attempted; and the impatience that is too apt to be felt at the seasonable and salutary delays of natural delivery. There is no standard period for the uterine expulsion of the fœtus. Various moral as well as physical causes are influential in either promoting or retarding parturition. It would, therefore, be presumptuous to decide, in any case, what should be the period of natural effort. The labour that, in certain circumstances of strength and energy, would be safely accomplished within twelve hours, in other cases, incapable of strong uterine contraction, would require four times that period to be either safe or seasonable. All this Nature understands: she operates by a nice adaptation of circumstances, which precision constitutes the state of necessity; and with this well-adjusted, and, in general, happily efficient order, there should be no interference, without the clearest persuasion of its being indispensably necessary.

Were I not restrained from relating instances of the mischievous effects resulting from the prevailing practice of man-midwifery, it would be abundantly in my power to adduce proofs of injury, that would greatly outweigh the cases stated by Mr. Atkinson of the indispensable necessity of scientific aid. It would be invidious and unfeeling to descend to particulars, where I conceive the principle on which the practice is founded is so open to attack, and so utterly utterly indefensable, as that which would authorise and vindicate the practice of man-midwifery in all cases, because, perchance, in one instance in a thousand, something like obstetric advice and management might be deemed requisite.

*Taunton; Jan.* 10, 1816.

